# Effects of Martial Arts Intervention in Children and Young People with Developmental Coordination Disorder (DCD): A Systematic Review

**DOI:** 10.3390/children13020282

**Published:** 2026-02-19

**Authors:** Beatriz Olhos, Marco Branco, Beatriz Rosa, David Catela, Cristiana Mercê

**Affiliations:** 1 Sport Sciences School of Rio Maior (ESDRM), Santarém Polytechnic University, Av. Dr. Mário Soares 110, 2040-413 Rio Maior, Portugal; 210500323@esdrm.ipsantarem.pt (B.O.); marcobranco@esdrm.ipsantarem.pt (M.B.); 210500280@esdrm.ipsantarem.pt (B.R.); catela@esdrm.ipsantarem.pt (D.C.); 2 Sport Physical Activity and Health Research & Innovation (SPRINT), Santarém Polytechnic University, Complex Andaluz, Apart 279, 2001-904 Santarém, Portugal; 3 Physical Activity and Health—Life Quality Research Centre (CIEQV), Polytechnique University of Santarém, Complex Andaluz, Apart 279, 2001-904 Santarém, Portugal; 4 Interdisciplinary Center for the Study of Human Performance (CIPER), Faculty of Human Kinetics, University of Lisbon, Cruz Quebrada-Dafundo, 1499-002 Lisboa, Portugal; 5 Quality Education—Life Quality Research Centre (CIEQV), Santarém Polytechnique University, Complex Andaluz, Apart 279, 2001-904 Santarém, Portugal

**Keywords:** children, motor development, motor learning, motor competence, coordination, martial arts, exercise, fitness, health

## Abstract

**Highlights:**

**What are the main findings?**

- Martial arts-based interventions were associated with significant improvements in children and adolescents with DCD, particularly in overall motor skills, balance, muscle strength, and coordination.
- No adverse effects were reported in the included studies, reinforcing the safety and applicability of martial arts (MA) practice as complementary intervention strategies in DCD.

**What are the implications of the main findings?**

- MAs can be integrated as a complementary therapeutic strategy in clinical and school settings for children with DCD.
- Professional supervision and progressive structuring of sessions are essential to ensure effectiveness and safety.

**Abstract:**

Background: Developmental Coordination Disorder (DCD) is a neurodevelopmental motor disorder characterised by marked difficulties in the acquisition and execution of motor skills, substantially affecting daily activities and quality of life. Martial arts (MAs), due to their multi-skilled nature, have been studied as possible intervention strategies to improve motor competence and functionality in children with DCD. Objectives: The present systematic review aimed to explore the effects of MA practice in children and adolescents with DCD, identifying the benefits, methodological characteristics and practical implications of existing interventions. Methods: The search was conducted in the PubMed, Web of Science, and EBSCO databases, following the PRISMA 2021 guidelines, using the keywords (developmental coordination disorder OR DCD OR dyspraxia) AND (karate OR judo OR taekwondo OR aikido OR martial art) AND (child OR preschool). Experimental and quasi-experimental studies that applied MA programmes to children and adolescents (≤18 years) with a confirmed diagnosis of DCD were included. Results: Of the 1834 identified records, five studies met the inclusion criteria. The MA modalities examined were karate, tai chi, and taekwondo. Across studies (n per study = 16–145), MA-based programmes consistently yielded significant pre- to post-intervention improvements in overall motor competence (MC), balance, muscle strength, and coordination; one study reported maintenance of coordination gains at 3-month follow-up. Methodological quality assessed with the Downs and Black checklist ranged from fair to good (scores = 18–22). No adverse events were reported. Conclusions: Based on the included studies, MA interventions demonstrate potential as an effective motor intervention approach for children and adolescents with DCD. Findings consistently indicated significant improvements in motor competence, balance, muscle strength, and coordination, with additional benefits observed in cognitive and psychosocial domains and no reported adverse effects.

## 1. Introduction

Developmental Coordination Disorder (DCD) is a neurodevelopmental motor disorder, characterised in the fifth edition of the Diagnostic and Statistical Manual of Mental Disorders (DSM-5) [1]. This condition significantly affects daily activities and may also compromise academic performance. Its prevalence is estimated at around 6% among school-aged children [2], occurring more frequently in boys than in girls [3].

DCD is diagnosed when the acquisition and execution of motor coordination skills are substantially below what is expected for the child’s chronological age [1,4]. These difficulties cannot be attributed to intellectual developmental disorders or visual impairments, nor are they the result of a neurological condition affecting movement. Diagnosis requires a multidisciplinary team, typically including a paediatrician or neurologist, a psychologist and a therapist or exercise professional trained in the application of the standardised motor assessment battery. In addition to the motor tests, the psychologist will assess the intelligence quotient, and other causes and disorders will also be screened with the intervention of the doctor. Whenever all these diagnostic requirements cannot be met, e.g., only motor tests are applied, the child cannot be diagnosed with DCD and is considered to have p-DCD (probable DCD) [1,4].

Despite its high incidence, making it the most common motor disorder in childhood, DCD has been described as a ‘hidden problem’ as it remains less studied and underestimated by health and education professionals compared to other neurodevelopmental disorders [2]. Children with DCD are often seen as simply “clumsy” or “awkward,” leading to their motor difficulties being interpreted as a lack of skill or commitment, rather than being recognised as clinical manifestations of the disorder. This widespread lack of knowledge contributes to the underestimation of the functional impact of DCD and hinders the diagnostic process, delaying access to appropriate interventions that could improve their participation in daily activities and their quality of life [5,6].

Children with DCD typically struggle with self-care tasks, such as using utensils for eating and dressing, and they face challenges in school contexts, particularly handwriting and participation in team sports [3,7]. These limitations, evident from an early age, can have significant repercussions on engagement across different life domains. These motor coordination difficulties lead to avoidance of age-appropriate physical and social activities. And, in turn, such avoidance reduces opportunities for social skill development and may compromise self-esteem, fostering feelings of isolation and exclusion [8,9]. Several studies indicate that children with DCD tend to be less physically active and exhibit lower fitness levels compared to their typically developing peers. Consequently, they present reduced cardiorespiratory fitness, higher risk of obesity, and increased adiposity [10,11].

Martial arts (MAs) have been practiced for thousands of years and are now predominantly practised as a sport, for self-defense and as a recreational activity [12]. Among the numerous forms of activities that are offered, MAs play an important role in children’s physical activity [13]. Although there are several MA disciplines, karate, taekwondo, kung fu, and judo are among the most practiced among practitioners [14]. The implementation of programmes based on various martial arts has been explored as a strategy to promote the development of motor skills and improve the physical condition of children, including children with p-DCD [12].

Research demonstrates positive effects of martial arts practice in children across multiple domains. Motor skill improvements include explosive strength, movement speed, agility, balance, and coordination. Beyond motor development, documented benefits include enhanced aerobic and anaerobic endurance, increased social skills and self-confidence, and decreased aggression. Furthermore, martial arts practice can positively influence mental health and character development in children [12].

The beneficial effects of practicing MAs in typically developing children are well documented in the existing literature, demonstrating the effectiveness of these practices. Considering the additional difficulties and challenges that children with DCD face, the benefits of practicing MAs may be especially important for this population, underscoring the need to explore this area of intervention. However, to date, no systematic review has addressed MA practice in children with DCD, representing a significant gap in current knowledge. Based on these premises, this systematic review aims to investigate and analyse the benefits of MA practice in children with DCD. The obtained results may contribute to the advancement of knowledge and the development of effective intervention strategies.

## 2. Materials and Methods

### 2.1. Protocol and Registration

This systematic review followed the latest version of the PRISMA guidelines [15]. The review protocol has been registered on the Prospero platform with ID 1141632.

### 2.2. Eligible Criteria

The research question for this systematic review was formulated using the PICOS framework, which for this study stands for the following: population—children and adolescents up to 18 years of age with DCD; intervention—martial arts programmes; comparisons—pre- and post-intervention assessments; outcomes—benefits of martial arts practice; study design—randomised controlled trials (RCTs) and experimental and quasi-experimental studies.

Based on this question, the following eligibility criteria were defined: (i) studies including children or adolescents with DCD or p-DCD; (ii) identification of DCD or p-DCD confirmed through one of the gold-standard motor tests, i.e., Movement Assessment Battery for Children (MABC) or Bruininks–Oseretsky Test of Motor Proficiency (BOT); (iii) studies involving formal martial arts practice, with at least one component supervised by a qualified instructor; (iv) studies including pre- and post-intervention assessments (e.g., motor coordination, balance, body composition, physical activity); (v) mandatory evaluation of at least one benefit of martial arts practice (e.g., improvement in motor coordination, balance, body composition, or physical activity levels); (vi) peer-reviewed scientific studies with RCT, experimental, or quasi-experimental design.

For this review, p-DCD referred to cases in which children presented motor performance significantly below what is expected for their age based on standardised tests yet where the complete multidisciplinary diagnostic assessment required for a formal DCD diagnosis was not feasible or not reported. This approach is common in paediatric research, particularly in intervention studies and in school contexts, where it is not always possible to operationalise all clinical procedures (e.g., assessment by a paediatrician and the administration of intelligence quotient tests by a psychologist), reflecting constraints related to access and the organisation of health services [6,16,17].

The inclusion of studies required confirmation of motor deficits using standardised and validated instruments, namely the Movement Assessment Battery for Children (MABC/MABC-2) or the Bruininks–Oseretsky Test of Motor Proficiency (BOT/BOT-2), which are widely recognised as “gold-standard reference” instruments for assessing motor competence in paediatric populations, ensuring diagnostic consistency and comparability across studies. Studies based exclusively on screening questionnaires or on non-standardised assessments were excluded to preserve methodological robustness and to reduce heterogeneity arising from the use of non-equivalent or non-validated measures.

### 2.3. Search Strategy and Databases

A comprehensive search of academic databases was conducted 5–8 February 2026. The databases used included PubMed, Web of Science, and EBSCO, with the following keywords: (developmental coordination disorder or dcd or dyspraxia) AND (karate OR judo OR taekwondo OR aikido OR martial art OR kickboxing OR muay thai OR sumo OR mixed martial arts OR kung fu) AND (child OR preschool). These databases were selected to ensure a broad coverage of biomedical, rehabilitation, and sport sciences research relevant to DCD. Given the focused nature of the research question targeting a single neurodevelopmental motor disorder (DCD) and a specific intervention approach, namely martial arts-based programmes, the search terms were intentionally specific. To enhance sensitivity across publication periods and terminological conventions, both contemporary (DCD) and historical terminology (dyspraxia) were included and specific modalities were combined (e.g., karate, judo, taekwondo, aikido) under the broader umbrella term “martial art”.

### 2.4. Study Selection

All retrieved articles were imported into Zotero for reference management. Duplicates were removed through comparative analysis of titles and abstracts by two authors independently. In addition, a manual screening was performed of the reference lists of all included studies to identify any further potentially relevant records; no additional eligible studies were found through this process. Study selection was performed independently by two reviewers at both stages: title/abstract screening and full-text eligibility assessment. Inter-rater reliability was excellent, with perfect agreement achieved at both stages (title/abstract: Cohen’s κ = 1.00; percent agreement = 100%; n = 1623; full-text: Cohen’s κ = 1.00; percent agreement = 100%; n = 6). No third-reviewer adjudication was required, as there were no discrepancies between reviewers’ decisions [18].

### 2.5. Data Extraction

The following information was extracted from each study: (i) authors; (ii) year of publication; (iii) title; (iv) country; (v) objective; (vi) sample; (vii) age; (viii) gender; (ix) type of intervention; (x) programme duration; (xi) equipment; (xii) type of exercise; (xiii) frequency; (xiv) intensity; (xv) session length; (xvi) supervision; (xvii) pre- and post-intervention results; and (xviii) main conclusions. In cases when papers did not contain all the information needed; the authors were contacted for more details.

### 2.6. Quality Assessment

Quality assessment was conducted independently by two reviewers using the Downs and Black (D&B) [19] quality rating scale. Disagreements were solved through discussion with a third reviewer. The D&B checklist scores range from zero to 28 points, with higher scores representing a higher quality. Previous studies used the following cut-off points to categorise the studies: excellent, 26–28; good, 20–25; fair, 15–19; poor, < 15 points [18].

## 3. Results

### 3.1. Studies’ Selection

**Figure 1:**
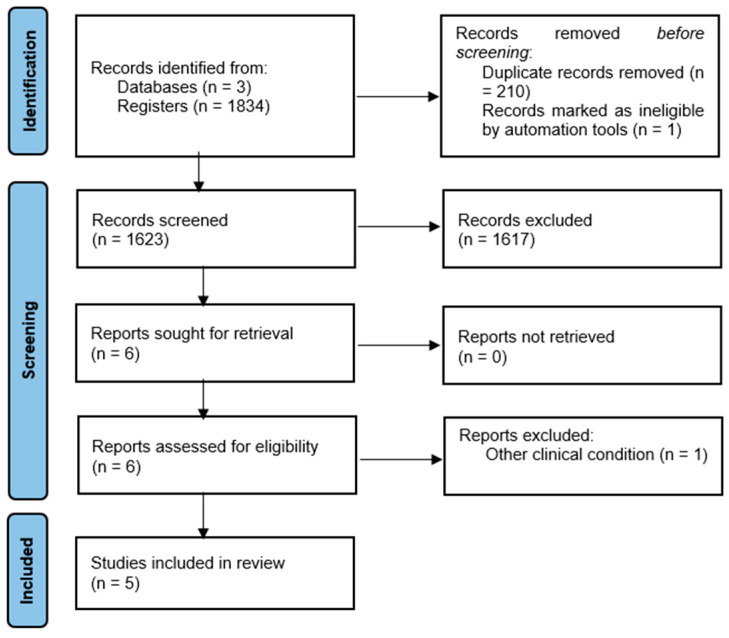
Flow diagram of the study selection process.

Initially, 1834 articles were identified through the search strategy applied in the databases PubMed, Web of Science, and EBSCO. After removing duplicates, studies whose titles and abstracts were not related to the topic of this review were excluded. Subsequently, the full texts of potentially eligible articles were analysed. After the complete screening of all articles, five studies met the inclusion criteria and were therefore selected for this systematic review (Figure 1). Inter-rater reliability between the two independent reviewers was perfect at both stages (title/abstract: κ = 1.00; full-text: κ = 1.00), with 100% agreement and no need for third-reviewer judgement.

All five included studies involved children and adolescents with formally diagnosed DCD according to the Diagnostic and Statistical Manual of Mental Disorders V. No studies with p-DCD samples were retained after full-text screening.

### 3.2. Characteristics and Main Results of the Studies

The present systematic review included five randomised controlled trials (RCTs) conducted between 2012 and 2022, which examined the effects of different martial arts modalities, such as karate [20], tai chi [21], and taekwondo [22,23,24], on children and adolescents with Developmental Coordination Disorder (DCD). All studies reported significant improvements in variables related to motor competence, muscle strength, balance, and coordination, although the magnitude and specificity of these effects varied according to the type and structure of the intervention.

A study by Ghadiri et al. [20], conducted in Iran, assessed the impact of an eight-week karate-do training programme in female adolescents, aged between 12 and 13 years, with DCD from different socioeconomic backgrounds. Each 75 min session included warm-up, Kihon practice (basic techniques), Kata (predetermined sequence of techniques that simulate combat against imaginary opponents), and Kumite (fight). Training intensity was qualitatively controlled and progressively increased throughout the programme by raising the complexity and physical demands of the techniques. In the post-intervention assessment, significant improvements were observed across all motor competence subtests (*p* < 0.001), particularly in fine motor integration (*p* < 0.009), manual dexterity (*p* < 0.020), bilateral coordination, and balance (*p* < 0.005). When analysing the socioeconomic backgrounds, participants from higher backgrounds demonstrated greater overall gains, suggesting that contextual factors may influence training response.

A study by Fong et al. [21], conducted in Hong Kong, compared the effects of a 12-week programme combining tai chi with muscle strength training (TC-MPT) versus tai chi alone, strength training alone, and a control group. The intervention included supervised in-person sessions and home practice: children attended weekly sessions led by qualified instructors, complemented by home exercises recorded by caregivers in a training diary to monitor adherence. After the intervention, the TC-MPT group showed significant improvements in dynamic balance (LOS), lower limb strength, and overall motor competence (MABC-2) (*p* < 0.01), as well as a reduction in falls (*p* < 0.05). Isolated programmes (TC or MPT) also yielded benefits, though to a lesser extent.

Two other studies by Fong et al. [22,23] (2012, 2013) explored different dimensions of taekwondo (TKD) in children with DCD. In the first [22], 12 weeks of TKD training resulted in significant improvements in postural control and sensory integration, reflected in increased Composite Equilibrium Score (SOT) and vestibular ratio (*p* < 0.05), as well as reduced body sway velocity (UST) (*p* < 0.05). These results indicate enhanced use of vestibular information to maintain balance, bringing the performance of children with DCD closer to that of typically developing peers. In the second [23], the focus was on isokinetic knee muscle strength and reactive and static balance. Training intensity was progressively adjusted throughout the 12-week programme by increasing movement complexity and raising kicking targets as children demonstrated technical and motor improvements. After three months of training, the TKD group exhibited a significant increase in knee extensor strength (+25.4%) and flexor strength (+33.6%) at 180°/s (*p* < 0.05), as well as a 60.6% reduction in unipedal sway velocity (*p* < 0.05), with no changes in reactive balance. These findings suggest that TKD promotes specific gains in muscle strength and static balance but does not influence reactive postural control.

Lastly, a study by Ma et al. [24], also conducted in Hong Kong, analysed an adapted taekwondo programme in prepubertal children, aged 6–9 years. After 12 weeks of training, the intervention group showed significant improvements in skeletal development (*p* < 0.001), a reduction in MABC impairment score (*p* < 0.016), and improved movement time in the eye–hand coordination test (*p* < 0.004). Differences in static balance and reaction time were not significant. The positive effect on coordination persisted at the three-month follow-up, suggesting long-term benefits for fine motor skills and bone development.

The main characteristics and results of the five analysed studies are present in Table 1 below.

Overall, all studies demonstrated positive effects of martial arts on motor competence and functional development in children and adolescents with DCD. Interventions combining components (tai chi + strength training) and those emphasising stability and muscle strength (karate and taekwondo) yielded the most comprehensive results. No adverse events were reported, reinforcing that adapted martial arts are safe and effective complementary strategies for improving motor skills in this population.

### 3.3. Characteristics of Interventions

The interventions analysed lasted between 8 and 12 weeks, with a frequency of predominantly three sessions per week, combining face-to-face sessions and home practice. The duration of the sessions varied between 60 and 90 min, with moderate intensity and gradual progression of technical demands (see Table 2).

All interventions were supervised by qualified instructors, although four studies included home training monitored by parent-completed logbooks [21,22,23,24]. In terms of the intervention content, the programmes integrated specific components of the martial arts modalities (e.g., Kihon and Kata in karate; tai chi movements associated with strength training; kicking and punching techniques in taekwondo), complemented by warm-up, stretching, and balance exercises.

## 4. Discussion

Although the benefits of martial art (MA) practice for children and adolescents are widely acknowledged [12,25], to date no systematic review has been conducted on this practice in children with DCD. In this sense, the present systematic review aimed to investigate and analyse the benefits of MA practice in children and adolescents with DCD. The results of the five included studies consistently demonstrated that MA-based interventions have a positive impact on motor skills in children and adolescents with DCD. Despite variation in martial arts modality, programme duration, frequency, and session structure most studies reported significant improvements in key motor outcomes including overall motor competence (MC), balance, muscle strength, and coordination. These results suggest that MAs, due to their integrative and multicomponent nature, may represent an effective intervention strategy for this population.

Beyond the observed improvements, several underlying mechanisms may help explain why MA interventions benefit children with DCD. From a motor learning perspective, MAs involve high levels of repetition, structured progression, and the development of increasingly complex motor schemas, which may facilitate the consolidation of new motor patterns [16,26,27]. The emphasis on bilateral movements, rapid postural transitions, and controlled force production likely enhances sensorimotor integration, improving the efficiency of proprioceptive and vestibular processing, all these domains are frequently impaired in DCD [15,28,29]. Additionally, the cognitive demands inherent to martial arts, such as sequencing, inhibition, attentional focus, and working memory, may stimulate executive control processes that support motor planning and coordination [30,31]. On a neurophysiological level, repeated practice of precise goal-directed movements may promote synaptic plasticity within cortico-cerebellar and cortico-striatal circuits implicated in motor learning [32,33]. Collectively, these mechanisms indicate that MAs function as a multicomponent intervention, simultaneously engaging motor, cognitive and sensorimotor domains, which may account for the wide range of improvements.

The studies analysed cover a 10-year period (2012–2022) and include only five interventions involving martial arts in this population. The enlarged search strategy, which retrieved 1834 records across three databases, further confirms that the scarcity of eligible studies reflects the limited state of research rather than insufficient search breadth. These data reflect, on the one hand, a sustained interest in the topic, but on the other, a scarcity of studies in this specific population, highlighting the need for further research. The diversity of assessment methodologies, ranging from screening instruments such as MABC, BOT, and DCDQ7 to various evaluation methods for distinct variables, reflects not only the heterogeneity of objectives but also the methodological approaches adopted. Future research should aim to standardise methodologies to facilitate meaningful comparisons across studies and different martial arts modalities.

It is also noteworthy that of the five studies we analysed, four were conducted in China and one in Iran, all within the Asian continent. This geographic distribution may reflect the cultural origins of several martial arts, including karate, kenpo, judo, and jiu-jitsu originating in Japan, taekwondo from South Korea, and tai chi from China. All these martial arts emerged from Asian culture, which may contribute to researchers and the general population being more familiar with their benefits and more inclined to apply them [34,35].

In comparative terms, the study by Ghadiri et al. [20], which analysed the effect of karate-do, highlighted improvements in all dimensions of MC assessed by BOT-2, with emphasis on bilateral coordination, balance, and strength. The work by Fong et al. [21] showed that the combination of tai chi with muscle strength training (TC-MPT) produced the most comprehensive benefits, including gains in dynamic balance, lower limb strength, and overall motor competence (MABC-2). The studies by Fong et al. [20,21] demonstrated that taekwondo training improved isokinetic strength and postural stability, while the study by Ma et al. [24] showed that adapted taekwondo may support skeletal development and eye–hand coordination.

Collectively, these results reinforce that MAs enhance multiple dimensions of motor control, providing stimuli for strength, balance, agility, and fine coordination. Regarding specific motor skills, all results reported significant improvements in at least one of the following areas: (i) overall MC [20,21], (ii) balance and postural control [21,22], (iii) lower limb muscle strength [23], and (iv) eye–hand coordination and fine motor skills [24]. These improvements reflect the controlled nature of MA exercises, which require postural adjustments, movement sequencing, and sensorimotor integration [12]. Thus, regardless of modality, evidence indicates that regular and supervised training contributes to improving fundamental motor skills and, potentially, to enhancing motor functionality in the daily lives of children with DCD. Although the number of studies per MA modality was very limited, and the heterogeneity of protocols prevents any modality-specific conclusions, some descriptive tendencies can be noted. Karate and taekwondo programmes [20,22,23,24], which typically emphasise strength, bilateral coordination, and postural control, were associated with improvements in muscle strength as well as fine and gross motor coordination. In contrast, the tai chi-based programme [21]—which, it is important to note, was implemented in combination with strength training—showed greater effects on dynamic balance, lower limb strength, and overall motor competence. These patterns should, however, be interpreted with caution, as differences in training structure, dosage, outcome measures, and sample characteristics do not permit direct comparison between modalities.

Such findings are consistent with the broader literature on motor interventions for DCD, which indicates that multicomponent approaches involving high repetition, structured practice, and progressive task demands tend to produce significant improvements in motor coordination, balance, and strength [29,36,37]. Task-oriented programmes, cognitive orientation to daily occupational performance, and neuromotor task training share core principles—functional practice, motor-learning mechanisms, and increased motor engagement—suggesting that martial arts can be conceptualised as an ecological and culturally relevant variant of motor interventions already validated in the literature.

Building on these findings, MAs also show promise as a structured and feasible component within multidisciplinary rehabilitation pathways for children and adolescents with DCD [16,17,37]. Their combination of task-oriented practice, graded progression, cognitive engagement, rapid postural control challenges and bilateral skill development aligns closely with core principles recommended in evidence-based motor interventions [28,35,36]. Moreover, MA programmes are inherently motivating, socially engaging, and relatively low-cost, which supports their potential integration into physiotherapy, occupational therapy, psychomotricity, and school-based rehabilitation settings [12,14,25]. From a practical standpoint, MAs can be considered a complementary therapeutic approach for children and adolescents with DCD in clinical or school settings. Their combination of structured movement, discipline, and playful elements may promote motivation, adherence, and self-confidence [12]. Interventions should be supervised by professionals with specific training to ensure safety, technical accuracy, appropriate intensity, and reliable programme implementation [14,21,22,23,24]. Future investigation should therefore explore how MA-based interventions can be systematically incorporated into existing therapeutic and school frameworks, including establishing safety protocols, therapist training, optimal dosage, and long-term functional outcomes [21,22,23,24,36,37].

This review also identified some methodological aspects that limit the generalisation of the results. In particular, the use of home-based training, reported on in the three studies by Fong et al. [21,22,23] and in Ma et al. [24], raises concerns about data reliability. Although these programmes included supervised in-person sessions, home-based training relied on parent-reported adherence without direct researcher supervision, introducing a potential risk of bias. Furthermore, the absence of objective intensity measures in several studies [20,23] limits comparability and hinders precise dose–response analysis.

It is important to acknowledge the limitations of this systematic review. Although recent studies with good methodological quality were included, the total sample of studies is small and geographically concentrated, predominantly in Asia. Overall study quality ranged from fair (n = 2) [20,23] to good (n = 3) [21,22,24], indicating acceptable rigor but also highlighting limitations on the strength and generalisability of the evidence. In particular, some methodological fragilities can be pointed to, such as small sample sizes, heterogeneity in programme and outcome measures, reliance on parent-reported adherence, and the limited use of intensity/load metrics in some studies. Taken together, these issues support a cautious interpretation of effect sizes while acknowledging that the consistent pattern of improvements in MC, balance and strength across all studies of fair to good quality increases confidence in the observed trends. Additionally, restricting the search to English-language publications may have excluded relevant studies in other languages [18].

Future research should address current gaps by standardising intensity and training load measurements (e.g., heart rate, perceived exertion, motion sensors) and by implementing more reliable methods for monitoring home practice, such as digital tools, mobile applications, or remote supervision.

## 5. Conclusions

The results of this systematic review indicate that martial arts (MAs) can be an effective and safe intervention for promoting motor development in children and adolescents with DCD. The analysed modalities, i.e., karate, tai chi, and taekwondo, demonstrated significant gains in MC, balance, muscle strength, and hand–eye coordination, emphasising the potential of these practices in motor rehabilitation. However, these findings should be interpreted cautiously due to the small number of studies, their fair to good methodological quality, and the heterogeneity in the interventions design and assessment methods.

Supervised interventions integrating combined and progressively structured exercises revealed greater positive impact, whereas programmes relying heavily on home-based training presented limitations on monitoring and reliability.

These findings reinforce the need to incorporate structured, supervised, and adapted MA programmes in clinical and school settings, contributing towards mitigating the motor consequences of DCD and improving motor development and social participation in daily activities for this population.

## Figures and Tables

**Table 1:** Main characteristics and findings of the five analysed studies.

Authors (Year)| Country|D&B Score	Aim	Sample	Intervention Results	Main Conclusions
Ghadiri et al. (2022) [20]|Iran|18	Evaluate 8-week intervention effects on MC in DCD adolescents from varying SEL	16 ♀ (12–13 y): 8 low SEL e 8 high SEL	↑ Total MC and subtests (*p* < 0.001); fine motor integration (*p* < 0.009); manual dexterity (*p* < 0.020); balance (*p* < 0.005); strength (*p* < 0.001)	Karaté significantly ↑ MC in both groups, with greater gains in the high SEL group
Fong et al. (2022) [21]|China|22	Compare the effects of tai chi + strength training (TC-MPT), tai chi alone, and control in DCD children	121 (9–12 y): TC-MPT—30 (25 ♂, 5 ♀) TC—30 (26 ♂, 4 ♀) MPT—30 (25 ♂, 5 ♀) CG—31 (25 ♂, 6 ♀)	TC-MPT: ↑ in LOS and MABC-2 (*p* < 0.01), strength (*p* < 0.01), sif. decrease in fall’s number (*p* < 0.05)	Combined training (CT-MPT) was the most effective for improving balance, strength, and MC
Fong et al. (2012) [22]|China|22	Evaluate the 3-month effect of TKD on sensory organisation and balance in DCD children	62 (6–9 y): DCD-TKD—21 (17 ♂ e 4 ♀) DCD-CG—23 (18 ♂ e 5 ♀) CG—18 (14 ♂ e 4 ♀)	DCD-TKD: ↑ in SOT and vestibular ratio (*p* < 0.05), ↓ in oscillation velocity in UST (*p* < 0.05) compared to DCD-CG, results similar to CG (*p* > 0.05)	DCD-TKD ↑ sensory integration and balance, especially the use of vestibular information
Fong et al. (2013) [23]|China|19	Evaluate the 3-month effect of TKD on knee strength and reactive and static balance in DCD children		DCD-TKD: ↑ in extensor strength (+25.4%) and flexor strength (+33.6%) (*p* < 0.05); UST body sway velocity sig. slower compared to DCD-CG (*p* < 0.001) and similar to CG (*p* > 0.05)	DCD-TKD ↑ strength and static balance, but not reactive balance
Ma et al. (2018) [24]|China|21	To evaluate the effect of adapted TKD on skeletal development and motor performance in prepubertal DCD children	145 (6–9 y; Tanner stage I): TKD—51 (45 ♂, 6 ♀) GC—94 (76 ♂, 18 ♀)	TKD: ↑ in skeletal developent (*p* < 0.001), MABC score (*p* < 0.016), and EHC (*p* < 0.004)	Adapted TKD accelerated skeletal development and improved eye–hand coordination; it did not influence static balance

D&B—Downs and Black checklist, ♀ — female sex, ♂ — male sex, y—years, ↑—improvement, ↓—decrease, MC—motor competence, SEL—socioeconomic levels, LOS—limits of stability, DCD—developmental coordination disorder, TKD—taekwondo, CG—control group, MABC—Movement Assessment Battery for Children, sig—significant, SOT—sensory organisation test, UST—single-leg balance test, EHC—eye–hand coordination.

**Table 2:** Intervention characteristics per reviewed study.

Authors (Year)	Program Duration	Assessments	Type of Exercise	Frequency/Intensity/Session Duration	Supervision
Ghadiri et al. (2022) [20]	8 weeks	**MABC2** and **DCDQ7**—DCD diagnosis **IPAQA**—PA level and Socio-economic questionnaire—to classify socio-economic level (high vs. low) **BOT-2**—MC	Kihon Hein Shodan Kata Warm-ups and stretching exercises	3×/week 75 min/session (30 min warming + 45 min karate) Progressive training	Yes
Fong et al. (2022) [21]	12 weeks	**BioSway**—assess stability limits **MABC-2**—measure MC **Lafayette Manual Muscle Test System** (Model 01165)—measure strength and time to maximum strength of knee extensors and flexors	Tai chi (TC) Resistance training (MPT) Combination of both modalities (TC-MPT)	3×/week (1 face-to-face + 2 home) 90 min/session TC—10× movement repetitions, lasting 5–10 min MPT—4 sets of 10 reps at 70% of 1RM TC-MPT—Combination of the above	Face-to-face session: physiotherapist and certified instructor (tai chi and fitness) Home sessions: parents with written instructions and logbook for recording progress
Fong et al. (2012) [22]	12 weeks	**Computerised Dynamic Posturography:** SOT, UST	Warm-up (light running, stretching) Punching and blocking techniques in a static stance Kicking techniques (front, side, circular, back), practiced in a combat stance Dynamic & static balance exercises. Relaxation and stretching	1× week in-person session + daily home sessions 60 min/session Moderate intensity: technique repetitions (20× punches/blocks, 40× kicks)	Face-to-face sessions: TKD instructors Home exercises: parents, with logbook monitored by researchers
Fong et al. (2013) [23]	12 weeks	**Cybex Norm isokinetic dynamometer:** strength of the knee extensors and flexors **Computerised Dynamic Posturography:** UST, MCT			
Ma et al. (2018) [24]	12 weeks	**Sunlight BonAge:** bone age **MABC:** MC **EHC:** accuracy, reaction time, movement time **Biodez Biosway:** static/postural balance	Adapted TKD class + daily practice at home with the same exercises. CG: jogging 1 h/day, monitored with a pedometer	1×/week face-to-face session + daily home sessions 60 min per session Moderate intensity, suitable for children: predefined repetitions (e.g., 20 punches/blocks, 40 kicks per technique)	Face-to-face sessions: black belt TKD instructor and one assistant Home sessions: parents (logbooks)

MABC—Movement Assessment Battery for Children; MC—motor competence; DCD—developmental coordination disorder; DCDQ7—Developmental Coordination Disorder Questionnaire; IPAQA—International Physical Activity Questionnaire for Adolescents; PA—physical activity; BOT-2—Bruininks–Oseretsky Test of Motor Proficiency; TC—tai chi, MPT + strength training; SOT—sensory organisation tests; UST—single-leg balance test; MCT—reactive equilibrium; TKD—taekwondo; CG—control group.

## Data Availability

No new data were created or analysed in this study.

## Abbreviations

The following abbreviations are used in this manuscript:

DCD	Developmental Coordination Disorder
p-DCD	Probable Developmental Coordination Disorder
MC	Motor Competence
SEL	Socioeconomic Level
BOT-2	Bruininks–Oseretsky Test of Motor Proficiency, Second Edition
MABC	Movement Assessment Battery for Children
MABC-2	Movement Assessment Battery for Children, Second Edition
DCDQ7	Developmental Coordination Disorder Questionnaire
IPAQA	International Physical Activity Questionnaire for Adolescents
TC	Tai Chi
MPT	Muscle Power Training
TC-MPT	Tai Chi combined with Muscle Power Training
TKD	Taekwondo
CG	Control Group
LOS	Limits of Stability
SOT	Sensory Organisation Test
UST	Unipedal Stance Test (single-leg balance)
MCT	Motor Control Test (reactive equilibrium)
EHC	Eye–Hand Coordination
RM	Repetition Maximum (used in strength training context)
D&B	Downs and Black checklist (quality assessment tool)
